# RETRACTION: Expression and Significant Roles of the lncRNA NEAT1/miR‐493‐5p/Rab27A Axis in Ulcerative Colitis

**DOI:** 10.1002/iid3.70192

**Published:** 2025-04-11

1

**RETRACTION:** H. Wang, J. Teng, M. Wang, Y. Zhang, X. Liu and Z. Liu, “Expression and Significant Roles of the lncRNA NEAT1/miR‐493‐5p/Rab27A Axis in Ulcerative Colitis,” *Immunity, Inflammation and Disease* 11, no. 5 (2023): e814, https://doi.org/10.1002/iid3.814.

The above article, published online on 16 May 2023 in Wiley Online Library (wileyonlinelibrary.com), has been retracted by agreement between the authors; the journal Editor‐in‐Chief, Marc Veldhoen; and John Wiley & Sons Ltd. The retraction has been agreed upon following an investigation into concerns raised by a third party. The investigation revealed that the LPS+lncRNA NEAT1‐sRNA+inhibitor control flow cytometry plot and parts of the LPS plot presented in Figure 5E have been published in other articles representing different scientific contexts. The authors provided an explanation and some data, but this was not deemed sufficient. The editors have lost confidence in the data presented and consider the conclusions to be compromised.

